# Electrically and Thermally Conductive Low Density Polyethylene-Based Nanocomposites Reinforced by MWCNT or Hybrid MWCNT/Graphene Nanoplatelets with Improved Thermo-Oxidative Stability

**DOI:** 10.3390/nano8040264

**Published:** 2018-04-22

**Authors:** Sandra Paszkiewicz, Anna Szymczyk, Daria Pawlikowska, Jan Subocz, Marek Zenker, Roman Masztak

**Affiliations:** 1 Institute of Materials Science and Engineering, West Pomeranian University of Technology, Piastow Av. 17, 70310 Szczecin, Poland; daria.pawlikowska@zut.edu.pl; 2 Institute of Physics, West Pomeranian University of Technology, Piastow Av. 48, 70311 Szczecin, Poland; anna.szymczyk@zut.edu.pl; 3 Department of Electrotechnology and Diagnostics, West Pomeranian University of Technology, Sikorskiego str. 37, 70310 Szczecin, Poland; jan.@zut.edu.pl (J.S.); marek.zenker@zut.edu.pl (M.Z.); 4 ELPAR Cable Factory, Laskowska str. 1, 21200 Parczew, Poland; roman.masztak@elpar.pl

**Keywords:** hybrid nanocomposites, thermal and electrical conductivity, carbon nanofillers, mechanical properties, thermo-oxidative stability

## Abstract

In this paper, the electrical and thermal conductivity and morphological behavior of low density polyethylene (LDPE)/multi-walled carbon nanotubes (MWCNTs) + graphene nanoplatelets (GNPs) hybrid nanocomposites (HNCs) have been studied. The distribution of MWCNTs and the hybrid of MWCNTs/GNPs within the polymer matrix has been investigated with scanning electron microscopy (SEM). The results showed that the thermal and electrical conductivity of the LDPE-based nanocomposites increased along with the increasing content of carbon nanofillers. However, one could observe greater improvement in the thermal and electrical conductivity when only MWCNTs have been incorporated. Moreover, the improvement in tensile properties and thermal stability has been observed when carbon nanofillers have been mixed with LDPE. At the same time, the increasing content of MWCNTs and MWCNTs/GNPs caused an increase in the melt viscosity with only little effect on phase transition temperatures.

## 1. Introduction

Polyethylene (PE) is found to be one of the most commonly used plastics in the world, belonging to the group of polyolefins [1]. Its wide applications, among others in the packaging, chemical, electrotechnical, and machine industries, result mainly from its easy processing, recyclability, and physical properties such as high wear resistance, high impact resistance, good chemical resistance, low density, physiological inactivity, and low price [2]. Additionally it does not absorb water [3]. Currently, in the cable industry, a broad range of polyolefins, alone or as blends, with various additives and conductive fillers are used as the materials to manufacture insulation and semi-conductive shields for the use in electric cables [4]. The typical construction of medium and high voltage cables includes a conductor surrounded by an insulation and semi-conductive layers/screens. The inner and intermediate semi-conductive layers are most often a semiconducting cross-linked polymer layer applied by extrusion around the conductive element and over the insulation layer. The insulating layer is selected from crosslinked polyethylene (XLPE), ethylene-propylene rubbers, and ethylene propylene diene rubbers (EPDM rubbers). A bonded or strippable semi-conductive shield in these cables is based on low density polyethylene (LDPE), linear low density polyethylene (LLDPE) or medium density polyethylene (MDPE) compositions with ethylene-vinyl acetate, ethylene alkyl acrylate or methacrylate copolymers, and with an appropriate type and amount of carbon black (CB) [4,5,6]. In order to achieve the electrical resistivity that will meet the requirements of cable standards (below 500 Ω·m), such composite has to be filled with large amount of CB (up to 40 wt. %) [6]. The results present herein are the part of wider project on obtaining PE based nanocomposites containing carbon nanofillers, such as graphene nanoplatelets (GNPs), carbon nanotubes (CNTs), which can be used as semi-conductive screens in medium voltage (MV) cables. It can be expected that such nanocomposites can exhibit higher conductivity at a lower filler loading than conventional semiconductive composites with conductive CB.

The field of polymer nanocomposite research is currently one of the most rapidly developing domains of materials science and engineering. So far, nanocomposites containing CNTs have sparked greater interest in comparison to graphene-based nanocomposites, which resulted mainly from the poor repeatability of the graphene derivatives (GDs) preparation techniques [7]. Graphene itself is a one atom thick, two-dimensional (2-D) sheet composed of sp^2^ carbon atoms arranged in a honeycomb lattice [8] with a carbon–carbon bond length equals to 0.142 nm [9,10]. Moreover, graphene exhibits various intriguing characteristics, such as high electron mobility at room temperature (250,000 cm^2^/Vs) [11,12], exceptional thermal conductivity (5000 W/mK in plane) [13], superior mechanical properties with Young’s modulus of ~1 TPa and ultimate strength of 130 GPa [12], an extremely high surface area (theoretical limit of 2630 m^2^/g), unique adsorption capability [14,15], and gas impermeability [16], which makes it the perfect candidate for improving electrical and thermal conductivity, along with mechanical, thermal and gas barrier properties of polymers [17,18]. However, uniform distribution of both CNTs and GDs throughout a polymer matrix, that leads to the improvement of functional properties (most importantly mechanical properties and electrical conductivity) is a cause for some concern in thermoplastic polymer matrices [19]. One of the latest solutions to solve the above mentioned problem is to utilize a mixture of CNTs and GDs in order to observe a so-called “synergistic effect” which in theory should result in a significant enhancement of properties of the obtained polymer nanocomposites. Several studies of hybrid system of CNTs and GDs that affect a variety of thermoplastic polymer matrices, such as PE [20,21,22,23], polypropylene (PP) [24], PET [25], poly(trimethylene terephthalate) (PTT) [26,27], poly(vinyl alcohol) (PVA) [28], (thermoplastic) elastomers [29,30,31,32,33], etc. have been already published. Significant enhancement of the carbon nanofiller dispersion was observed along with the improvement in electrical properties of the multi-phase composites [34]. Generally, preventing nanofiller’s agglomeration leads to a “synergistic effect” on the properties of polymer composites, and the re-agglomeration during polymerization aids in achieving a lower percolation threshold in the composite [30].

In our previous work [35], we have presented how the addition of GNPs affect the selected properties of LDPE. While in this paper, LDPE-based nanocomposites with hybrid system of nanofillers comprising of CNTs and GNPs were prepared in order to enhance the dispersion quality of the melt blended materials, thus improving electrical and thermal conductivity, thermal stability and mechanical characteristics. The functional properties of the obtained hybrid nanocomposites were thoroughly studied and favorably compared with those of neat LDPE and those containing only CNTs, or previously published GNPs. Taking into consideration the possible potential application of semi-conductive LDPE based nanocomposites in the cable industry, for fabrication of nanocomposites, the multi-walled carbon nanotubes (MWCNTs) and GNPs with technical quality for industrial application were used. Herein, common industrial processing technique (melt processing) has been applied for preparation of LDPE based nanocomposites which is essential for mass production.

## 2. Materials and Methods

### 2.1. Materials

Low density poly(ethylene) (LDPE) (Malen E FGAN 23-D003, Lyondell Basell, Bayreuth, Germany), has been applied as polymer matrix in the obtained nanocomposites, with the density of 0.922 g/cm^3^ and melt flow rate (MFR 190 °C/2.16 kg) of 0.31 g/10 min. As nanofillers one used: Nanocyl^®^ NC7000™ series, thin multi-walled carbon nanotubes (MWCNTs) produced via the Catalytic Chemical Vapor Deposition (CCVD) process, with the average diameter of 9.5 nm; average length of 1.5 μm; purity of 90%; surface area of 250–300 m^2^/g; volume resistivity of 10^−4^ Ω∙cm, and GNPs (Grafen^®^-iGP2, Grafen Chemical Industries, Ankara, Turkey) for technical applications, with the thickness of ~5–10 nm (ca. 30 sheets), average diameter of 5–10 μm; purity < 99%, I_D_/I_G_ rate (according to Raman spectroscopy) equals to 0.08 and volume resistivity of 0.65 Ω∙cm.

### 2.2. Preparation of LDPE-Based Nanocomposites

Nanocomposites based on LDPE contacting MWCNTs, or the hybrid system of MWCNTs/GNPs (has been melt blended using twin screw extruder (LSM30, Leistritz Laboextruder, Nuremberg, Germany) with closely occurring scrolls and interchangeable mixing sections, (diameter: *D* = 34 mm, *L*/*D* ratio = 23) equipped with two dispensers, a cooling bath and a granulator. One prepared three series of LDPE-based nanocomposites:

Nanocomposites containing MWCNTs with the concentrations of: 1.5, 3, 5, 7, 10, and 20 wt. %;

Nanocomposites containing hybrid system of MWCNTs:GNPs 1:1 with the total concentration of nanofillers: 3, 5, and 20 wt. %;

Nanocomposites containing hybrid system of MWCNTs:GNPs 3:1 with the total concentration of nanofillers: 3, 5, and 20 wt. %.

In each case, at the beginning, one prepared the masterbatch (with the concentration of 20 wt. % of nanofiller/nanofillers) which was then diluted to lower concentrations in the LDPE matrix. The following parameters were determined for extruding the masterbatch: feed zone: 20 °C; zone 1: 100 °C; zone 2: 170 °C; zone 3: 180 °C; zone 4: 190 °C; zone 5: 200 °C; zone 6: 210 °C; zone 7: 220 °C; and zone 8: 20 °C (nozzle). Rotational speed of screws of 40 rpm for masterbatches and nanocomposites containing of 4–10 wt. % of carbon nanofillers and of 150 rpm for nanocomposites containing of 1.5, 3 wt. % of MWCNT was used. Yield: 1.5 kg/h.

The used processing conditions of LDPE/MWCNTs composites were established by evaluation of electrical resistivity measurements supported by SEM analysis of LDPE/MWCNTs nanocomposites. Moreover, the parameters used in this study allow to obtain high repeatability of the conductivity results (Table 1), which were also confirmed during extrusion of screens on the actual technological line within implementing research works in the project. The results obtained within the project dedicated to LDPE/CNTs nanocomposites showed that electrical resistivity/conductivity (Table 1) at concentrations below and above 3 wt. % of CNTs in LDPE was influenced by the speed of the extruder’s screw. The LDPE/CNTs nanocomposites showed that rotational speed of twin screw extruder of 40 rpm for composites containing above 3 wt. % of CNTS in a small error range gave the repeatable values of resistivity/conductivity of LDPE based composites.

### 2.3. Preparation of Testing Samples

Samples for electrical conductivity measurements were formed at the temperature of 190 °C and under pressure 5 bar (for 1 min) and 10 bar (for 1 min) by compression molding (Colin P200E, Dr COLLIN GmbH, Ebersberg, Germany) to the form of polymer foils with the thickness of ~250 μm. The thickness of thin foils was measured with a Micrometer mod. 293–521 from Mitutoyo. Five measurements were taken for each sample, with an experimental error of ±0.001 mm. The thickness is an average value.

The dumbbell shape samples (type A3, PN-ISO 37) for tensile, density and thermal conductivity measurements were obtained by injection molding using Boy 15 (Dr BOY GmbH & Co., Neustadt-Fernthal, Germany) injection molding machine. The parameters of injection molding were determined following the guidelines of PN-EN ISO 294 standard, and the melting points of the materials determined from differential scanning calorimetry (DSC). The cut out sections of the samples were used for thermal analyses (DSC, TGA). The samples for SEM analysis were cryofractured in liquid nitrogen and subsequently coated (2–5 nm) in vacuum with a thin gold film before the tests. Before performing the measurements all samples were conditioned in accordance to PN-EN ISO 291 + AC1 standard.

### 2.4. Characterization Methods

The dispersion of MWCNTs and the hybrid system of MWCNTs and GNPs in the LDPE matrix was observed using SEM (JEOL JSM 6100, Freising, Germany).

The electrical conductivity of the obtained nanocomposites was evaluated by measurements of resistivity using Electrometer 6517A (Keithley Instruments, Inc., Germering, Germany) device together with a set of Keithley 8009. The measurements were performed accordingly to the following standards:

(a) for resistivities lower than 10^4^ Ω cm according to the standard PN-EN ISO 3915;
(b) for resistivities higher than 10^4^ Ω cm according to the standard PN-88/E-04405.

The thermal conductivity coefficient of the polymer/nanocomposites, was determined using the transient plane source (TPS) method, the Hot Disk TPS 2500 S (Uppsala, Sweden), and the Hot Disk thermal constants analyzer. The measurements were performed according to ISO 22007-2. Three tests were conducted and the mean values were reported for each nanocomposite material.

Differential scanning calorimetry (DSC) measurements were carried out using a DSC TA Q100 instrument under a nitrogen atmosphere in a temperature range from −25 to 200 °C at the heating and cooling rate of 10 °C/min and sample weight of 10 ± 0.2 mg. Each DSC testing cycle consisted of heating, cooling and 2nd heating scan. The first cooling and second heating scans were used to determine the melting and crystallization peaks. The heat fusion has been determined by integration of the normalized area of melting endotherm. The degree of crystallinity of the sample (*X*_c_) was calculated using following equation:

(1) $$X_{c}\;=\;\Delta H_{m}/\Delta H_{m}^{0}\left(1\;-{\;\varphi }_{n}\right)$$

where $\Delta H_{m}^{o}$ is the enthalpy change of melting for a 100% crystalline sample, for PE equals to 293 J/g [36], ϕ*_n_* is a weight content of nanofiller, and $\Delta H_{m}$ ∆Hmis derived from melting peak area on DSC thermogram.

Density (*d*) measurement was performed using a hydrostatic scales (Radwag WPE 600C, Radom, Poland), calibrated using working standards of known density. For each sample, the density value was calculated as the mean of five specimens.

The melt viscosity of the samples was measured using ARES rheometer (Rheometric Scientific Inc., New Castle, DE, USA). The measurement was performed at 200 °C in frequency range 0.1–50 Hz, in a parallel-plate fixture (diameter = 25 mm) with a gap distance of 2 mm.

The tensile properties of the samples were measured using Autograph AG-X plus (Shimadzu, Duisburg, Germany) tensile testing machine equipped with a 1 kN Shimadzu load cell, an non-contact optical extensometer and the TRAPEZIUM X computer software, operated at a constant crosshead speed of 5 mm/min. Measurements were performed at room temperature with the grip distance of 20 mm. According to PN-EN ISO 527 standard, the tensile modulus, tensile strength and elongation at break of the nanocomposites were determined. Five measurements were conducted for each sample, and the results were averaged to obtain a mean value.

Thermo-oxidative stability of the obtained materials was performed using thermogravimetry analysis (TGA 92-16.18 Setaram, Caluire-et-Cuire, France) using the system to measure the simultaneous TG-DTG. Measurements were carried out in an oxidizing atmosphere i.e., dry, synthetic air (N_2_:O_2_ = 80:20 vol. %). The study was conducted in the temperature range of 20–700 °C at the heating rate of 10 °C/min. Measurements were performed in accordance with the PN-EN ISO 11358:2004 standard.

## 3. Results and Discussion

### 3.1. Morphology of LDPE-Based Nanocomposites

**Figure 1:**
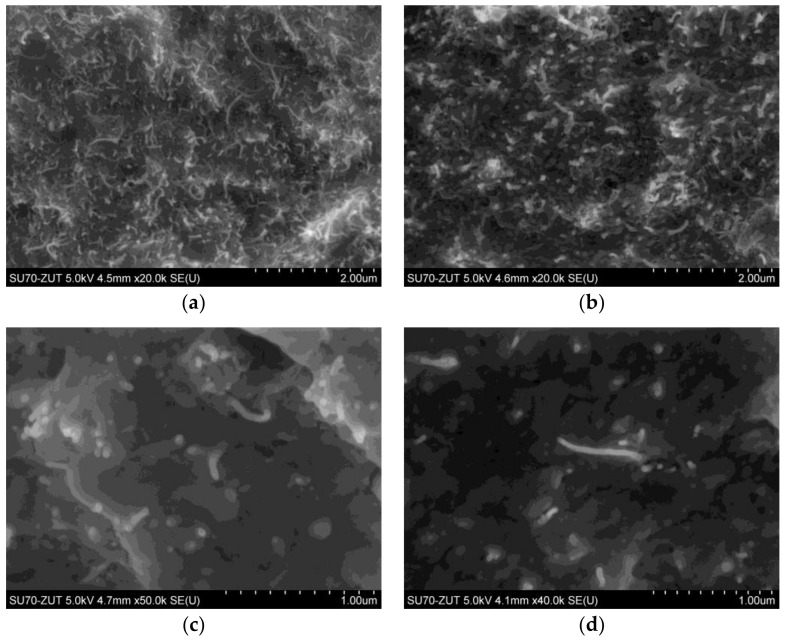
Scanning electron microscopy (SEM) images of: (**a**) LDPE/20 wt. % MWCNTs; (**b**) LDPE/10 wt. % MWCNTs; (**c**) LDPE/5 wt. % MWCNTs; and (**d**) LDPE/1.5 wt. % MWCNTs.

It has been already investigated that the structure and morphology of polymer nanocomposites is strongly related to the fabrication process. In general, three methods are used to disperse CNTs and/or GDs into the polymer matrix, i.e., in situ polymerization, solution blending and melt blending. The first two ones, usually result in good dispersion while the melt blending causes poor dispersion [37,38]. However, both, in situ polymerization and solvent blending, require large amounts of organic solvent, which is poisonous and not convenient for industrial processing [37,39]. SEM images of the fracture surfaces for the LDPE/MWCNTs nanocomposites are shown in Figure 1. One can observe, that at both, higher (10 and 20 wt. %) (Figure 1a,b) and lower (≤5 wt. %) (Figure 1c,d) MWCNTs contents, the LDPE/MWCNT nanocomposites exhibited a relatively uniform dispersion of the MWCNTs in the LDPE matrix. Moreover, some MWCNT bundles were pulled out from the LDPE matrix, and this indicated that some of the nanotube bundles were individually dispersed in the polymer matrix (especially Figure 1c,d). In general, short CNTs with high aspect ratio, and large surface area are often subjected to self-agglomeration or bundle formation at higher concentrations and thus easily form interconnected or network-like structures in the molten polymer matrix [40,41]. Nevertheless, herein the carefully selected processing parameters (extrusion) have enabled obtaining nanocomposites with an appropriate degree of distribution of CNTs even at the content of 20 wt. %.

**Figure 2:**
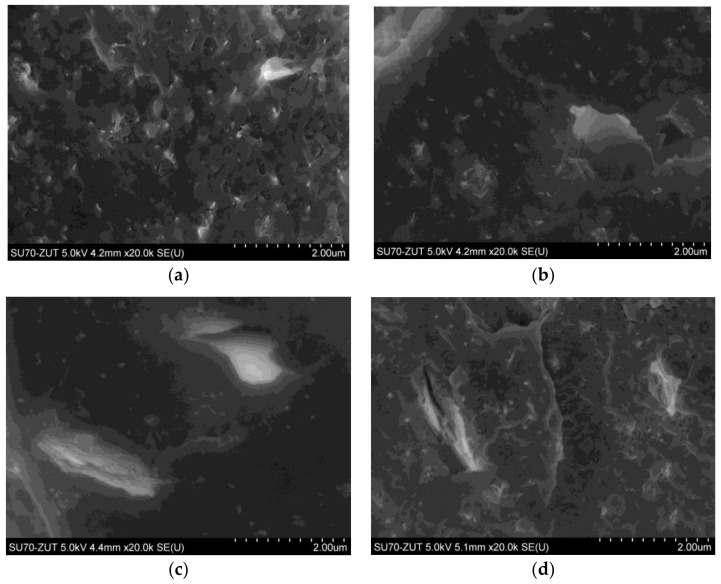
SEM images of: (**a**) LDPE/5 wt. % (MWCNTs:GNPs 3:1); (**b**) LDPE/3 wt. % (MWCNTs:GNPs 3:1); (**c**) LDPE/5 wt. % (MWCNTs:GNPs 1:1); and (**d**) LDPE/3 wt. % (MWCNTs:GNPs 1:1).

The SEM images of LDPE-based hybrid nanocomposites with the MWCNTs to GNPs ratios of 3:1 and 1:1 are presented in Figure 2. Low magnification micrographs (Figure 2a,c) show that both MWCNTs and GNPs are reasonably distributed within the polymer matrix. In turn, higher magnification micrographs (Figure 2b,d) reveal how MWCNTs and GNPs interlocate to form a local percolation networks. Moreover, Figure 2b confirms that GNPs are not perfect disk-shaped nanosheets, which probably resulted from the shear-mixing forces that could have broken them into smaller fragments and distort their shapes [42]. Whereas, CNTs (Figure 2a,c) play the role of interconnects to form a percolation network between the GNPs and thus act as conductive flexible pathways spanning in-between the larger high-surface area GNPs. Since, these two different shaped nanoparticles cooperate synergically, one expects to observe the enhancement of the electrical and thermal conductivities along with mechanical and thermal properties of the hybrid nanocomposites.

### 3.2. Electrical and Thermal Conductivity

**Figure 3:**
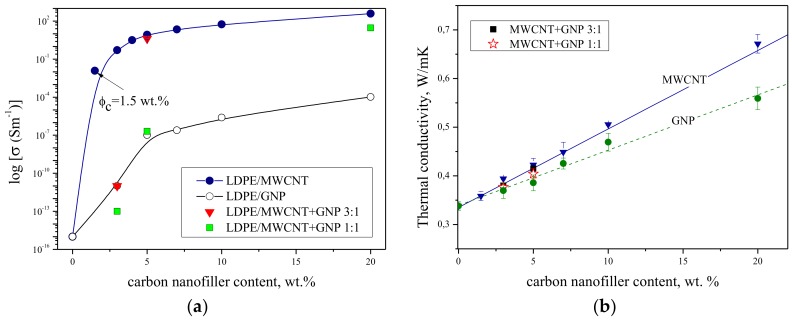
(**a**) The electrical conductivity and (**b**) the thermal conductivity vs. nanofiller content (wt. %) of LDPE/MWCNTs, LDPE/GNPs and LDPE/MWCNTs + GNPs nanocomposites.

Figure 3 shows that electrical (a) and thermal conductivity (b) of LDPE-based nanocomposites increase with the increasing content of carbon nanofillers in the matrix. Both, electrical and thermal conductivity measurements reveal that one can achieve greater improvement in conductivity when the proper dispersion is obtained, i.e., the separation into single nanoparticles, which is one of the critical issues with regard to the processing of polymer nanocomposites due to the small size and high aspect ratio, leading to the formation of agglomerates or aggregates of nanoparticles [43]. As shown in Figure 3a, a significant increase in electrical conductivity for about thirteen orders of magnitude was observed with only 1.5 wt. % of MWCNTs (percolation threshold φ_c_). The obtained value is lower than those reported by He et al. [44] with φ_c_ = 2 wt. % for the high density polyethylene (HDPE)/MWCNTs composites prepared by solution-precipitation, or by Zhang et al. [45] with φ_c_ = 4 wt. % for those prepared by melt processing, or by Ciselli et al. [46] with φ_c_ = 3.1 wt. % for those prepared by solution casting-drawing using mixed-solvents. While, Du et. al. [20] for HDPE/MWCNTs nanocomposites with a segregated network structure obtained the percolation threshold of about 0.15 vol. % (0.32 wt. %). Herein, with further increase of MWCNTs content only slight improvement in electrical conductivity was visible. In turn, GNPs didn’t provide such a sharp increase in electrical conductivity, and the φ_c_ equals about 5 wt. % [35]. In fact, Xie et al. [47] provided a theoretical study devoted to compare the effective conductivity of the composites reinforced by graphene nanosheets (GNSs) and CNTs, which has shown that the composites reinforced by GNSs have a lower percolation threshold and higher conductivity and critical exponent, and can form a conductive network more easily than those reinforced by CNTs with the same volume fraction. However, our observations are more similar to those made by Du et al. [20], who obtained for HDPE/GNSs nanocomposites the percolation threshold of 1 vol. % (2.13 wt. %), which was more than six times higher than those obtained for HDPE/MWCNTs nanocomposites. Furthermore, LDPE/MWCNTs nanocomposites exhibit higher electrical conductivity (six orders of magnitude higher) than LDPE/GNPs nanocomposites with the same nanofiller weight fraction, implying that MWCNTs are a more efficient conductive nanofiller. This in fact is in agreement with the observations made for LDPE/MWCNTs + GNPs hybrid nanocomposites. The masterbatch (20 wt. % of total content of nanofillers) has only a slightly lower value of electrical conductivity in comparison to LDPE/20 wt. % MWCNTs. The subsequent dilutions to 5 wt. %, and then 3 wt. %, (MWCNTs + GNPs) show that the hybrids with the ratio of 3:1 MWCNTs:GNPs exhibit higher values of electrical conductivities in comparison to the one with the MWCNTs:GNPs ratio of 1:1. Moreover, both hybrids at the concentration of 3 wt. % have even lower values of conductivity if compared even to LDPE/3 wt. % GNPs nanocomposite. It is hard to unambiguously explain why the hybrids of MWCNTs with GNPs didn’t provide greater enhancement in electrical conductivity. The conduction mechanism of nanocomposite is very complicated. One can find that many factors can affect the electrical properties. In order to prepare a useful conductive nanocomposites containing MWCNTs and/or GNPs with high electrical conductivity and low percolation threshold, one should take into consideration several crucial issues, i.e., development of more effective composite preparation method (or modify melt blending technique), avoiding CNTs agglomeration and GNPs rolling under shear force during processing etc.

The above observations are in good agreement with the thermal conductivity results. The thermal conductivities of the obtained LDPE-based nanocomposites measured by the hot disc method are depicted in Figure 3b. The CNTs and graphene sheets have exceptional high thermal conductivity [48,49,50]. Non-defected single graphene sheet at room temperature has thermal conductivity of 5300 W/m·K in plane and relatively low out-of-plane around 2 W/m·K [49]. The out-of-plane thermal coupling is limited by week van der Waals interactions. Thermal conductivity of GDs decreases with increasing their thickness, approaching the value of the bulk graphite limit. By comparison, natural graphite exhibits thermal conductivity ranging from 100 to 400 W/m·K [50]. For fabrication of LDPE nanocomposites one uses available GNPs for industrial applications with the thickness of ~5–10 nm. As mentioned above, the thermal transport in the two-dimensional graphene is affected by its size (aspect ratio) and number of graphene layers (*n*).

In the present study, the addition of MWCNTs into the LDPE matrix caused greater improvement in thermal conductivity. The lower values of thermal conductivities was seen when GNPs were mixed with the matrix, while the hybrid consisted of MWCNTs and GNPs exhibited the values of thermal conductivity in between those obtained for “single” nanocomposite. This probably resulted from the rule of mixture, and unfortunately no synergistic effect was obtained. Several studies that deal with the use of two or more fillers with different shapes that may be beneficial in term of thermal conductivity enhancement, usually concern single-walled carbon nanotubes (SWCNTs). For instance, a synergistic effect between GNPs and SWCNTs in the enhancement of the thermal conductivity of epoxy composites was reported by Yu et al. [51], ascribed to the formation of a more efficient percolating hybrid CNT/GNP network with significantly reduced thermal contact resistance. Similar results obtained King et al. [52,53] for PP composites, in which thermal conductivity was synergistically enhanced by combining CNTs with carbon black and synthetic graphite. In turn, Im and Kim [54] in epoxy-based nanocomposites containing hybrid system of GO/MWCNT observed that the maximum thermal conductivity was obtained with the addition of about 0.36 wt. % of MWCNT to the filler. Then, it decreased with increased MWCNT loading but still remained higher than the thermal conductivity of the GO/epoxy composite. The measured thermal conductivity was compared with the results of a theoretical approach based on the effective medium approximation (EMA) developed by Maxwell-Garnett (M-G).

The hybrid system of MWCNT and GNPs didn’t provide synergistic enhancement to the electrical and thermal conductivity (Figure 3). This may result from the presence of a thin (few nm) layer of polymer (Figure 1 and Figure 2), which prevents the direct contact between the MWCNTs and GNPs and introduces a scattering layer for and phonons’ transport as well as an insulating layer in the tunneling barrier for electrical transport [55,56]. According to theoretical modeling [55,56], an increase in the thickness of the polymer layer from 0 to 10 nm does not affect significantly the heat transport, however such an increase of the width of the tunneling barrier would effectively eliminate the electrical transport. However, in the present study, despite receiving proper dispersion, the improvement in the electrical and thermal conductivity resulted from the increasing content of the nanofillers, with no synergistic effect.

### 3.3. Structural and Rheological Characteristics

**Figure 4:**
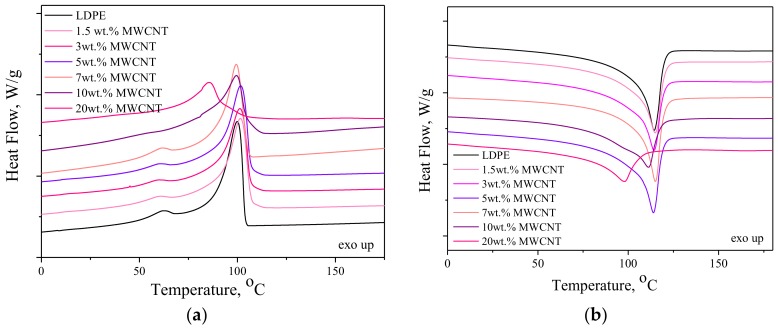
Differential scanning calorimetry (DSC) thermograms for LDPE/MWCNT nanocomposites recorded during cooling (**a**) and second heating (**b**).

**Figure 5:**
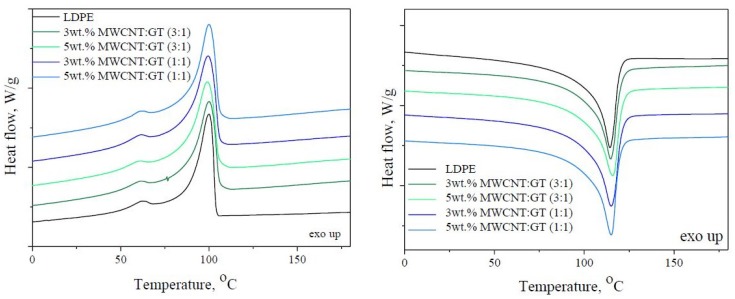
DSC thermograms for LDPE/MWCNT + GNP hybrid nanocomposites recorded during cooling (**a**) and second heating (**b**).

The nonisothermal crystallization behavior of LDPE, LDPE/MWCNT, and LDPE/MWCNT + GNP nanocomposites were studied using DSC. The DSC thermograms recorded during cooling from 200 to −25 °C and heating up are presented in Figure 4 and Figure 5, for LDPE/MWCNT and LDPE/MWCNT + GNP nanocomposites, respectively. Additionally, Table 2 lists the phase transition temperatures and corresponding enthalpies of melting and crystallization, degree of crystallinity and hydrostatic density of LDPE-based nanocomposites. The cooling curves show that neat LDPE crystallized at 100 °C and the incorporation of MWCNT did not significantly change the crystallization temperature (*T*_c_) (Figure 4a). The lowest value of *T*_c_ has been observed for LDPE/20 wt. % MWCNT, where the shift toward lower temperatures equals to 15 °C. Generally, an increase of *T*_c_ is attributed to the heterogeneous nucleation induced by MWCNTs. Herein, for the nanocomposite with the highest concentration of MWCNTs an antinucleating behavior was observed. In turn, from the second heating curve (Figure 4b) one can find that neat LDPE undergoes crystal melting at 114 °C, while the melting points (*T*_m_) of nanocomposites containing 1.5–10 wt. % of MWCNT did not change much, being recorded at the range of 111–115 °C. However, similarly as observed above, the lowest value of *T*_m_ was observed for LDPE containing 20 wt. % of MWCNT. Moreover, the incorporation of MWCNTs caused a decrease in degree of crystallinity, wherein the lowest value of *X*_c_ was observed for the nanocomposite containing 20 wt. % of MWCNT. The observed reduction in *X*_c_ differs from some of the reported data [57], in which case an increase of polymer crystallinity was claimed by adding CNTs. However, it is in the agreement with the studies of Kodjie et al. [58], who suggested that the reduction of the crystallinity was due to the fact that CNTs can break the continuity of the polymer matrix and large, uniform lamellae could not have been formed. Moreover, Pavlidou and Papaspyrides [59] reported that in thermoplastic nanocomposites, plate-like particles indicated a reduction of degree of crystallinity at higher contents.

Furthermore, the incorporation of both nanofillers at the ratios of 3:1 and 1:1 also didn’t cause any influence on crystallization and melting temperatures. One can only observe that when two types of nanofillers were added a slight decrease in enthalpies of crystallization and melting were observed. However, at the same time all hybrids exhibited comparable values of degree of crystallinity to one another and to neat LDPE. Moreover, all nanocomposites, demonstrated higher values of density in comparison to LDPE, which resulted from the fact that the density of both nanofillers (~2.1 g/cm^3^) is higher than the polymer matrix (~0.93 g/cm^3^). The obtained results are in agreement with the observations made by Wegrzyn et al. [60] for PP/MWCNTs + GNP hybrid nanocomposites. They noticed that the reduced confinement of polymer chains in the presence of agglomerated MWCNTs and GNPs restricted the formation of perfect crystals. Therefore, at high nanofillers content along with the presence of agglomerates, one can observe the distraction of the crystallites quality which affects the phase transition. This gave a significant reduction of melting temperature up to 3.8%. Moreover, Tarani et al. [61] indicated that the addition of GNPs in HDPE decelerated the overall crystallization process of matrix. GNPs behave as a barrier during the mass transfer forming a tortuosity in the diffusion path, which decreases the free volume and retard the crystalline-amorphous interface movement [62,63].

**Figure 6:**
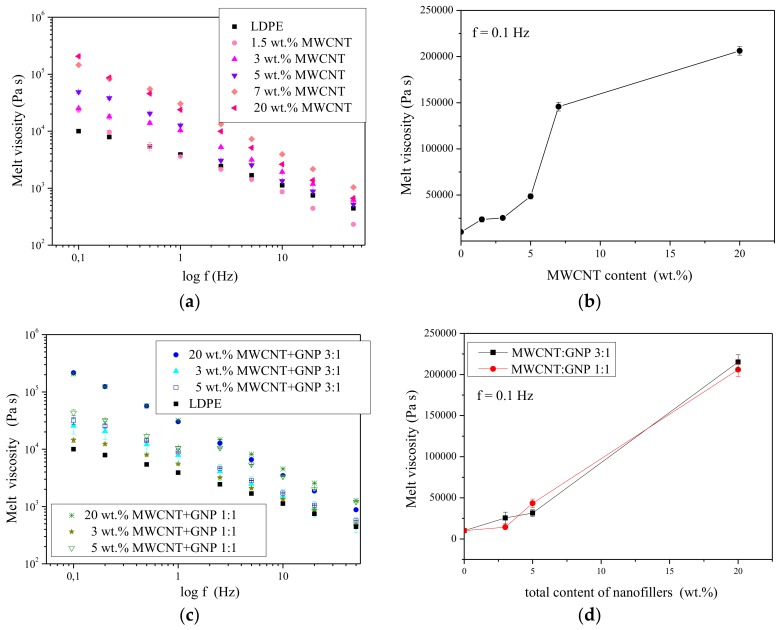
Melt viscosity as a function of frequency (**a**) and nanofiller content (**b**) for LDPE/MWCNT nanocomposites and melt viscosity as a function of frequency (**c**) and total nanofiller content (**d**) for LDPE/MWCNT + GNP hybrid nanocomposites.

Additionally, the rheological behavior of LDPE-based nanocomposites has been studied and the melt viscosity as a function of frequency and nanofillers’ content has been plotted in Figure 6. Rheological behavior of polymer nanocomposites in the molten state plays a critical role in understanding processability and structure–property relationships for these materials. On the other hand, melt rheology measurements can probe behavior of the relatively large material that is crucial from the macroscopic point of view [64]. Herein, the sharp increase of melt viscosity along with the decreasing frequency (Figure 6a) and increasing concentration of MWCNTs (Figure 6b) has been observed. Similarly, the increasing content of the mixture of two nanofillers that differ in shape affect the melt viscosity of LDPE (Figure 6c,d). The same higher values of melt viscosity were observed for the system with MWCNT:GNP ratio of 3:1 (Figure 6c). However, it also depended on the total concentration of nanofillers (Figure 6d). Moreover, as the rheological behavior is usually affected by polymer-polymer interactions, nanofiller-nanofiller interactions (CNT-CNT, CNT-GNP, and GNP-GNP) and polymer-nanofiller interactions [65,66] among others, one can conclude that the both MWCNT and GNP provided polymer-to-nanofiller interactions. It is also on the agreement with our previous observations made for PTT/MWCNT-COOH + GNP hybrid nanocomposites [67].

### 3.4. Thermo-Oxidative Stability

**Figure 7:**
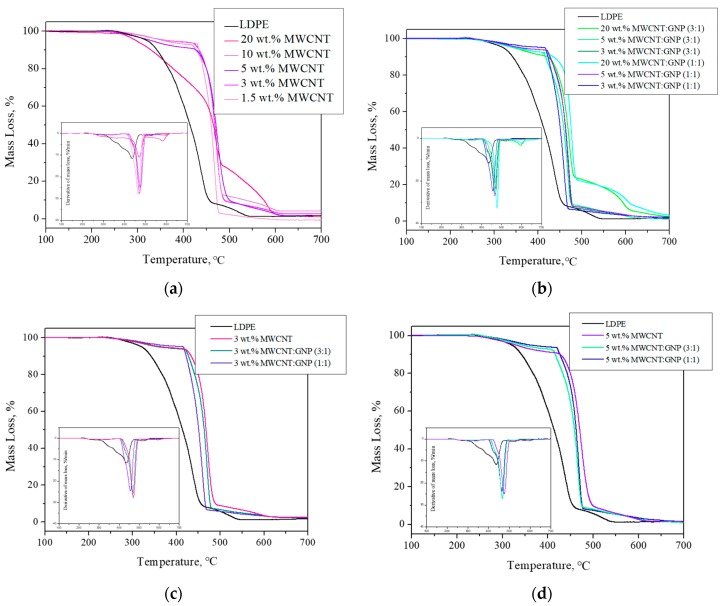
Mass loss and derivative of mass loss curves for: (**a**) LDPE/MWCNT nanocomposites; (**b**) LDPE/MWCNT + GNP hybrid nanocomposites; (**c**) LDPE-based nanocomposites at the total nanofiller content of 3 wt. %; and (**d**) LDPE-based nanocomposites at the total nanofiller content of 5 wt. %, measured in an oxidizing atmosphere.

Generally, the addition of carbon nanofillers, like CNTs or GNPs can improve thermal and thermo-oxidative stability of polymer matrices [22,25,33,34,35,36]. Figure 7a presents the thermal degradation behavior of LDPE/MWCNTs nanocomposites in air. The results show that the thermo-oxidative degradation temperature of LDPE/MWCNTs nanocomposites at a maximum of mass loss are from 436 °C to 466 °C and 479 °C for LDPE, LDPE/3 wt. % MWCNT and LDPE/20 wt. % MWCNT, respectively. Moreover, the addition of as little as 1.5 wt. % of MWCNTs caused a shift of a maximum of mass loss of about 27 °C toward higher temperatures. The results clearly indicate that the incorporation of carbon nanotubes will raise the temperature of maximum thermo-oxidative degradation. Similar observations were made for LDPE/MWCNT + GNP hybrid nanocomposites (Figure 7b). In every single case the addition of carbon nanoparticles improved thermo-oxidative stability of LDPE matrix. The most significant improvement was observed for the highest total concentration of nanofillers (i.e., 20 wt. %), however, already 3 wt. % of MWCNT:GNP at the ratios of 3:1 and 1:1 allowed us to observe the improvement of thermo-oxidative stability of the obtained nanocomposites. In addition we have compared how nanotubes and nanoplatelets affect the thermal oxidative stability at the same total concentration. Therefore, we have plotted the mass loss and derivative of mass loss curves for LDPE-based nanocomposites at the content of 3 and 5 wt. % (Figure 7c,d). In both cases, the greatest improvement was observed when only MWCNTs were added. The mixture of both, MWCNTs and GNPs, didn’t provide synergistic effect. Probably, it resulted from the not very high quality of GNPs, which is intended for technical applications. However, in our previous study [35], it was found that the incorporation of 3–10 wt. % of GNPs into LDPE caused a gradual increase in the thermo-oxidative temperature of the polymer matrix of about 20 wt. %. Therefore, perhaps lack of the interactions between MWCNTs and GNPs in annihilating free radicals, makes in this case, the nanotubes themselves, to be a more promising nanofiller, which is in the agreement with our previous study on the effect of SWCNTs and/or GNPs on the thermal stability of poly(trimethylene terephthalate-block-poly(tetramethylene oxide) segmented copolymer [33]. However, it is worth paying attention to the fact that GNPs (500 EUR/1kg) were characterized by a much higher price than MWCNTs for industrial application (75–95 EUR/1kg), so perhaps in summary, a mixture of two types of nanoparticles may be more attractive.

### 3.5. Tensile Properties

**Figure 8:**
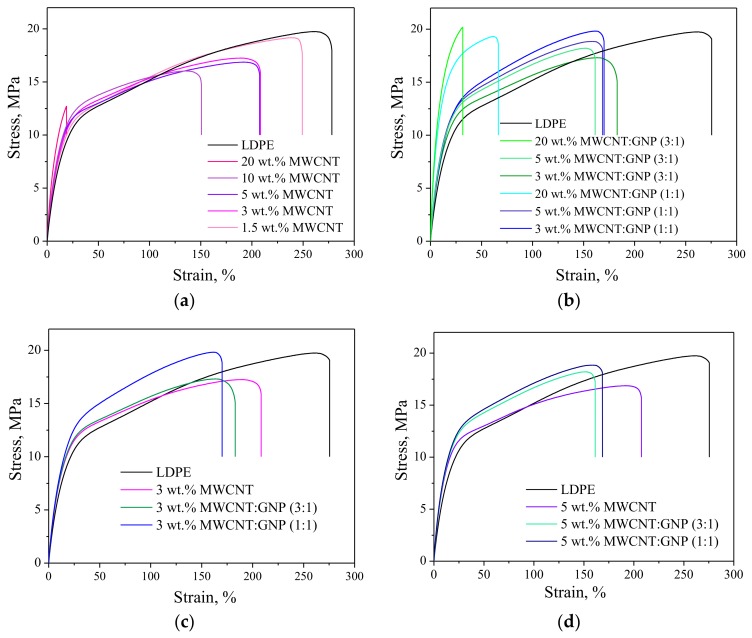
Stress-strain curves for (**a**) LDPE/MWCNT nanocomposites; (**b**) LDPE/MWCNT + GNP hybrid nanocomposites; (**c**) LDPE-based nanocomposites at the total nanofiller content of 3 wt. %; and (**d**) LDPE-based nanocomposites at the total nanofiller content of 5 wt. %.

Figure 8 indicates the tensile properties of LDPE-based nanocomposites. It was found that the addition of carbon nanofillers, like MWCNTs and/or GNPs increase initial tensile properties of the nanocomposite (Figure 8a). The values of Young’s modulus significantly increase along with the addition of MWCNT from 137 MPa, to 150 MPa, and 200 MPa, for LDPE, LDPE/5 wt. % and LDPE/20 wt. % of MWCNT, respectively (Table 3). It means that the incorporation of 20 wt. % caused an improvement of Young’s modulus of about 50%. Similarly, in the case of hybrid nanocomposites also sharp increase in Young’s modulus values was visible. In this case, the greatest improvement was obtained for LDPE/5 wt. % of MWCNT:GNP 1:1, and it was over 10 MPa higher that for LDPE/5 wt. % of MWCNT:GNP 3:1. This is best seen when comparing stress-strain curves at the same nanoparticles’ concentrations (Figure 8c,d). However, along with the increase of nanoparticles concentration, the decrease in tensile strength and elongation at break were observed. The lowest values of σ_m_ and ε_b_ were observed for LDPE/20 wt. % of MWCNT. This confirms our previous study on LDPE/GNP nanocomposites [35]. Furthermore, this is in the agreement with the observations made by McNally et al. [68], who prepared PE/MWCNT nanocomposites with compositions ranging from 0.1 to 10 wt. % by melt blending, reported a poor stress transference between the matrix and the filler. They argued that the mechanism of reinforcement is based on interfacial interactions between the matrix and the filler. In addition, She et al. [69] also observed that the tensile strength of HDPE/expanded graphite (EG) composite increased with increasing filler content up to 4 wt. % followed by a reduction. They indicated that the graphite nanosheets behave like a rigid particle and the conducting network formed with the EG content in the range 4–6 wt. % reduced significantly the mobility of the HDPE molecules such that they could not dissipate external applied mechanical energy. Consequently, the reduction of the tensile strength of the composites could not be avoided.

## 4. Conclusions

The aim of this study was polymer nanocomposites based on LDPE containing MWCNTs or the mixture of MWCNTs and GNPs. The developed procedure along with processing parameter allows to obtain nanocomposites with desired functional properties. The obtained LDPE-based nanocomposites exhibited high degree of homogeneity in the whole volume of polymer matrix, regardless of the amount of the used nanofiller or the mixture of two nanofillers that differ in shape. In addition, this method, due to its simplicity, is suitable for the use in an industrial scale. DSC studies showed that the addition of less than 10 wt. % of MWCNTs or MWCNT + GNP caused no effect on the melting and crystallization temperatures. Only, when 20 wt. % of MWCNT were added into LDPE a sharp decrease of melting and crystallization temperatures was visible, which was due to the fact that CNTs restrict the mobility of LDPE chains causing the difficulty in forming crystalline lamellae. The thermo-oxidative stability has been significantly improved. Moreover, the thermal conductivity of LDPE/MWCNTs and LDPE/MWCNT + GNP nanocomposites has been also improved, which could have been caused mainly by the addition of highly thermal conducting MWCNT and or/GNPs along with a small increase in degree of crystallinity. In addition, all nanocomposites were found to be electrically conducting. A sharp increase in electrical conductivity for about thirteen orders of magnitude was observed with only 1.5 wt. % of MWCNTs, which was a percolation threshold. However, no synergetic effect was achieved when a mixture of two types of nanofillers were incorporated into LDPE matrix. Mechanical properties, such as tensile strength and elongation at break, deteriorated slightly, which could be associated with reduced mobility of LDPE chains (therefore they could not dissipate external mechanical energy). In turn, Young’s modulus values increased significantly as a result of the addition of MWCNTs or the mixture of MWCNTs and GNPs.

## Figures and Tables

**Table 1:** Electrical resistivity of low density polyethylene/multi-walled carbon nanotubes (LDPE/MWCNTs) nanocomposites fabricated at different speeds of the extruder’s screw (rpm).

Content of MWCNT wt. %	Resistivity */Ω·m		
	at 150 rpm	at 60 rpm	at 40 rpm
1.5	(8.1 ± 2.1) × 10^3^	8.4 × 10^4^	1 × 10^6^
3	12.0 ± 7.5	27.0 ± 17	1.94 ± 12
4	-	0.36 ± 0.04	0.32 ± 0.04
5	1.22 ± 0.76	0.12 ± 0.07	0.12 ± 0.04
20	(0.3 ± 0.01) × 10^−2^	-	(0.26 ± 0.01) × 10^−2^

* Resistivity measured for nanocomposites fabricated at different rotational speed of extruder’s screw (rpm).

**Table 2:** Phase transition temperatures and corresponding enthalpies of melting and crystallization, degree of crystallinity and hydrostatic density of LDPE-based nanocomposites.

Sample	*T*_m_ [°C]	Δ*H*_m_ [J/g]	*T*_c_ [°C]	Δ*H*_c_ [J/g]	*X*_c_ [%]	*d* [g/cm^3^]
LDPE	114	128.9	100	129.1	44.0	0.934 ± 0.002
LDPE/1.5 wt. % MWCNT	115	120.2	101	120.3	41.7	0.942 ± 0.001
LDPE/3 wt. % MWCNT	114	112.6	101	112.2	39.6	0.951 ± 0.001
LDPE/5 wt. % MWCNT	114	119.1	102	119.4	42.7	0.962 ± 0.002
LDPE/7 wt. % MWCNT	114	104.8	100	104.8	38.4	0.968 ± 0.001
LDPE/10 wt. % MWCNT	110	104.1	100	101.3	39.4	0.985 ± 0.003
LDPE/20 wt. % MWCNT	97	102.1	85	102.7	38.7	0.934 ± 0.002
LDPE/3 wt. % MWCNT:GNP (3:1)	115	126.4	100	122.8	44.5	0.953 ± 0.001
LDPE/5 wt. % MWCNT:GNP (3:1)	116	121.8	99	121.6	43.8	0.962 ± 0.001
LDPE/3 wt. % MWCNT:GNP (1:1)	115	127.7	100	123.5	44.9	0.951 ± 0.003
LDPE/5 wt. % MWCNT:GNP (1:1)	115	124.0	100	122.5	44.5	0.961 ± 0.006

*T*_m_—melting temperature, *T*_c_—crystallization temperature, Δ*H*_m_ and Δ*H*_c_—enthalpies of melting and crystallization, respectively, *X*_c_—degree of crystallinity, *d*—density.

**Table 3:** Mechanical properties of neat LDPE and LDPE-based nanocomposites.

Sample	E [MPa]	σ_m_ [MPa]	ε_b_ [%]
LDPE	137.5 ± 3.30	19.77 ± 0.51	280.87 ± 8.16
LDPE/1.5 wt. % MWCNT	144.30 ± 8.07	19.03 ± 0.13	240.61 ± 26.39
LDPE/3 wt. % MWCNT	144.25 ± 6.65	17.94 ± 0.42	166.20 ± 10.66
LDPE/5 wt. % MWCNT	150.09 ± 8.06	16.76 ± 0.24	223.81 ± 11.25
LDPE/10 wt. % MWCNT	170.53 ± 7.19	15.90 ± 0.17	142.40 ± 6.76
LDPE/20 wt. % MWCNT	201.26 ± 8.80	12.57 ± 0.63	19.01± 2.78
LDPE/3 wt. % MWCNT:GNP (3:1)	158.78 ± 5.40	17.07 ± 0.21	187.05 ± 5.40
LDPE/5 wt. % MWCNT:GNP (3:1)	167.91 ± 3.29	18.06 ± 0.27	171.2 ± 13.59
LDPE/3 wt. % MWCNT:GNP (1:1)	164.32 ± 3.48	19.46 ± 0.31	178.4 ± 18.84
LDPE/5 wt. % MWCNT:GNP (1:1)	177.78 ± 5.09	18.79 ± 0.33	163.3 ± 10.77

E—Young’s modulus; σ_m_—tensile strength; ε_b_—elongation at break.
